# One statistical test is sufficient for assessing new predictive markers

**DOI:** 10.1186/1471-2288-11-13

**Published:** 2011-01-28

**Authors:** Andrew J Vickers, Angel M Cronin, Colin B Begg

**Affiliations:** 1 Department of Epidemiology and Biostatistics Memorial Sloan-Kettering Cancer Center 1275 York Avenue, Box 44, New York, NY 10065 USA; 2 Center for Outcomes and Policy Research Dana Farber Cancer Institute Boston, Massachusetts USA

## Abstract

**Background:**

We have observed that the area under the receiver operating characteristic curve (AUC) is increasingly being used to evaluate whether a novel predictor should be incorporated in a multivariable model to predict risk of disease. Frequently, investigators will approach the issue in two distinct stages: first, by testing whether the new predictor variable is significant in a multivariable regression model; second, by testing differences between the AUC of models with and without the predictor using the same data from which the predictive models were derived. These two steps often lead to discordant conclusions.

**Discussion:**

We conducted a simulation study in which two predictors, *X *and X*, were generated as standard normal variables with varying levels of predictive strength, represented by means that differed depending on the binary outcome *Y*. The data sets were analyzed using logistic regression, and likelihood ratio and Wald tests for the incremental contribution of X* were performed. The patient-specific predictors for each of the models were then used as data for a test comparing the two AUCs. Under the null, the size of the likelihood ratio and Wald tests were close to nominal, but the area test was extremely conservative, with test sizes less than 0.006 for all configurations studied. Where X* was associated with outcome, the area test had much lower power than the likelihood ratio and Wald tests.

**Summary:**

Evaluation of the statistical significance of a new predictor when there are existing clinical predictors is most appropriately accomplished in the context of a regression model. Although comparison of AUCs is a conceptually equivalent approach to the likelihood ratio and Wald test, it has vastly inferior statistical properties. Use of both approaches will frequently lead to inconsistent conclusions. Nonetheless, comparison of receiver operating characteristic curves remains a useful descriptive tool for initial evaluation of whether a new predictor might be of clinical relevance.

## Background

In determining whether a patient does have or will have a medical condition or outcome - respectively, diagnosis and prognosis- doctors can generally make use of clinical or laboratory data than are of proven predictive value. It has been argued that, when evaluating a novel predictor, the key question is whether it offers superior predictive accuracy to these established variables [1,2]. For example, the risk of cancer recurrence is known to be associated with clinical characteristics such as tumor size, cancer stage, lymph node involvement and possibly other factors. Any evaluation of the merit of, say, a new biomarker needs to take into account the abilities of the known clinical predictors. An appropriate study of this issue involves the comparison of the accuracy of a predictive model including only the established clinical variables with the accuracy of a model that included both the established variables and the new marker.

The advent of countless novel biomarkers in medical research has led to a large literature of studies seeking to test the value of these new markers as predictors of medical outcomes. Because of widespread concerns about the dangers of inappropriate uses of statistical methods in this setting, many prominent methodologists and subject matter experts have published articles seeking to provide advice on how to conduct these studies[1-6]. There is broad agreement that it is of value to determine incremental discriminative ability of the new marker, using comparisons of the area under the receiver operating characteristic (AUC) curve or c-index[1] of the predictive model with and without the new marker. Although all of these experts stop short of advocating that discrimination be compared using widely available tests for comparing AUCs [7,8], the advocacy of this type of comparison seems to have led many investigators to believe that such formal comparisons are indeed the recommended approach. Our article addresses the propriety of this strategy.

## Discussion

We define r(*X*) as a predictive model for a disease state *D *in which *X *corresponds to one or more established clinical and biological markers. If X* designates a new marker, our goal is to assess whether X* adds to the predictive accuracy that is already available from *X*. Thus, our question about the value of X* involves a comparison of r(*X*) to r(X, X*) rather than a comparison of r(*X*) to r(X*). That is, we wish to know the incremental increase in accuracy due to X*. The setting in which one wants to consider the addition of a new marker to an existing set of markers is known as a nested model.

There are two general approaches that are used frequently to compare the incremental effect of a new predictor in the context of a nested model. In the first, the novel marker is added to a regression model that includes the established markers. The new marker is accepted as having predictive value if it is significantly associated with outcome in the multivariable model, after adjusting for established markers; if so, the marker is commonly referred to being an "independent" predictor of outcome. In the second approach, the area under the receiver operating characteristic (ROC) curve (AUC) or c-index is calculated for each of the two predictive models, r(*X*) and r(X, X*), and the two AUC's compared. The fact that expert commentaries on this issue advocate this comparison without reference to statistical testing suggest that they have intended the comparison to represent an informal judgment of the increase in incremental accuracy[2-6], that is, the recommendation is to use a comparison of AUC's for estimation. Nonetheless, there are well known and widely-used statistical tests for comparing diagnostic accuracy[7,8], and so increasingly investigators have elected to use these as formal tests for incremental accuracy in the context of comparing predictive models[9-14].

We have observed that it is often the case in such reports that the novel marker has been found to be statistically significant in the multivariable model including established markers, but has been reported to have a non-significant effect on predictive accuracy on the basis of tests comparing the two AUCs. For instance, Folsom et al.[15] looked at various novel markers to predict coronary heart disease, and tested whether these markers added incremental value to a standard predictive model that included age, race, sex, total cholesterol, high density lipoprotein, blood pressure, antihypertensive medications, smoking status, and diabetes. For each marker, the authors reported both the *p *value for the marker in the multivariable model, and a *p *value for the difference in AUC between the standard model and the standard model plus the new marker. As an example, interleukin 6 was reported to be a statistically significant independent predictor of outcome (*p*=0.03), but the increase in AUC, from 0.773 to 0.783 was reported to be non-significant. Similarly, Gallina and colleagues [16] investigated whether body mass index could help predict high-grade prostate cancer. They reported that although body mass index was statistically significant (*p*=0.001) in a multivariable model including clinical stage, prostate volume, and total and free prostate specific antigen, the increase in AUC (from 0.718 for the standard predictors to 0.725 for the standard predictors plus body mass index) was non-significant (*p*=0.6).

In considering the contribution of a new marker in the context of established markers we are interested in the incremental improvement in predictive accuracy that the new marker can deliver. What do we mean by incremental predictive accuracy? A new predictor can only provide additional information if it is associated with the outcome, conditional on the existing predictors. Consequently, we are fundamentally interested in testing for conditional independence between the new predictor, X*, and the outcome, *Y*, conditional on the established predictors, *X*. If X* and *Y *are associated, conditional on *X*, then there is information that can be potentially utilized to improve the prediction. In other words, in constructing a test for incremental information, the conceptual null hypothesis is that there is no useful information in X* for predicting *Y *once the information in *X *is taken into account.

In the construction of a specific statistical test, the actual null hypothesis used can differ, even though in our context all tests are targeted fundamentally at the preceding conceptual null hypothesis. When we approach the question of the value of X* using a regression model, such as logistic regression if the outcome is a binary event or proportional hazards regression for survival-type outcomes, we are comparing the fit of the data to two different models, a null regression model in which the outcome, after transformation, has a linear relationship with *X *versus a model in which the addition of a linear term involving X* improves the fit. If *β *is the parameter representing the coefficient of X* in this model, then the null hypothesis is that *β=0*. This might lead to a different result from, say, a Mantel-Haenszel test of association between X* and *Y*, stratified by *X*. However, both are essentially testing the same conceptual null hypothesis, the hypothesis that there is no conditional association between X* and *Y*, given *X*, and thus no potentially useful incremental information in X* for the purposes of predicting *Y*.

Consider now approaching this issue in the setting of an ROC analysis. Again, there are different options for formulating the null hypothesis. A logical choice is to construct a test of the hypothesis that the ROC curve mapped by the predictor from the model r(*X*) is identical to the ROC curve mapped by the predictor from the model r(X, X*). Indeed tests of this nature are available [17]. However, by far the most common approach is to focus on the areas under the ROC curves from these two models [8]. The null hypothesis is that the areas, denoted AUC(*X*) and AUC(X,X*), are identical. These two null hypotheses are not the same, but they both conform to our conceptual null hypothesis, namely that X* does not add incremental information to the predictive ability of the model formed using *X *alone. Investigators who have used this approach have typically taken the patient-specific risk predictors from the two models, and used these as data elements both for estimating the ROC curves and as data for conducting the test comparing ROC areas.

To our knowledge, little work has been done to estimate the power of regression models for detecting incremental predictive accuracy in comparison to the power of corresponding tests for the AUCs. We conducted a simulation study in which the two predictors, X and X*, were generated as standard normal variables with varying levels of predictive strength, represented by means that differed depending on the binary outcome Y. The difference in means between Y = 1 and Y = 0 for X and X* are represented by μ and μ*respectively and were varied between 0 (i.e. the null) and 0.3. X and X* were generated both independently (i.e. with a correlation of ρ = 0.0) and for the correlations ρ = 0.1, ρ = 0.3 and ρ = 0.5. The data sets were analyzed using logistic regression, and likelihood ratio and Wald tests for the incremental contribution of X* were performed. The patient-specific predictors for each of the models were then used as data for a test comparing the two AUCs, using the popular area test proposed by Delong et al. [8]. The algorithm used for the simulation is provided in the Appendix. The results for a study with n=500 and an outcome prevalence of 0.5 are presented in Table 1. The first set of rows represent test size, i.e. the setting in which X* contributes no incremental predictive accuracy (represented by μ*=0). Here we see that both the likelihood ratio and Wald test have test size close to the nominal 5%. By contrast the DeLong test of the AUCs is exceptionally conservative, with a test size far below nominal. Power comparisons in the rest of the table show that the likelihood ratio test and the Wald test have similar power but both are far superior to the AUC test. Further, the likelihood and Wald tests are largely unaffected by the underlying strength of the baseline predictive model (represented by μ), while the power of the area test diminishes as the underlying AUC increases (again represented by μ). Power for all tests increases with greater correlation between μ and μ*.

**Table 1:** Simulation results for n=500, prevalence at 20%.

μ*	μ	Test	ρ=0.0	ρ=0.1	ρ=0.3	ρ=0.5
0.0	0.0	LRT	0.050	0.048	0.053	0.055
		
		Wald	0.048	0.045	0.052	0.053
		
		AUC	0.004	0.004	0.006	0.003
	
	0.1	LRT	0.059	0.057	0.053	0.052
		
		Wald	0.057	0.055	0.052	0.051
		
		AUC	0.003	0.004	0.002	0.005
	
	0.2	LRT	0.048	0.054	0.055	0.049
		
		Wald	0.045	0.051	0.054	0.047
		
		AUC	0.002	0.004	0.001	0.000
	
	0.3	LRT	0.043	0.059	0.052	0.054
		
		Wald	0.043	0.056	0.051	0.051
		
		AUC	0.002	0.001	0.000	0.002

0.1	0.0	LRT	0.217	0.196	0.215	0.259
		
		Wald	0.211	0.191	0.213	0.253
		
		AUC	0.027	0.025	0.040	0.043
	
	0.1	LRT	0.191	0.218	0.232	0.239
		
		Wald	0.187	0.212	0.226	0.237
		
		AUC	0.027	0.024	0.029	0.035
	
	0.2	LRT	0.199	0.203	0.198	0.253
		
		Wald	0.196	0.196	0.195	0.249
		
		AUC	0.021	0.015	0.019	0.022
	
	0.3	LRT	0.179	0.205	0.200	0.259
		
		Wald	0.178	0.205	0.199	0.253
		
		AUC	0.011	0.013	0.012	0.018

0.2	0.0	LRT	0.613	0.615	0.646	0.738
		
		Wald	0.607	0.613	0.643	0.734
		
		AUC	0.196	0.195	0.229	0.294
	
	0.1	LRT	0.614	0.621	0.650	0.736
		
		Wald	0.611	0.618	0.644	0.729
		
		AUC	0.167	0.178	0.203	0.272
	
	0.2	LRT	0.604	0.620	0.640	0.740
		
		Wald	0.600	0.616	0.637	0.735
		
		AUC	0.121	0.121	0.141	0.205
	
	0.3	LRT	0.595	0.623	0.641	0.696
		
		Wald	0.590	0.620	0.637	0.692
		
		AUC	0.096	0.098	0.111	0.153

0.3	0.0	LRT	0.908	0.926	0.942	0.970
		
		Wald	0.908	0.925	0.941	0.969
		
		AUC	0.581	0.586	0.622	0.745
	
	0.1	LRT	0.918	0.915	0.939	0.973
		
		Wald	0.916	0.913	0.936	0.972
		
		AUC	0.533	0.539	0.592	0.699
	
	0.2	LRT	0.910	0.925	0.933	0.971
		
		Wald	0.908	0.922	0.931	0.970
		
		AUC	0.414	0.432	0.496	0.627
	
	0.3	LRT	0.905	0.900	0.937	0.972
		
		Wald	0.903	0.898	0.936	0.970
		
		AUC	0.359	0.362	0.423	0.520

Entries in the table show the power (for μ* > 0) and the test size (for μ*=0) for the likelihood ratio (LRT) and Wald tests from logistic regression and the Delong et al. test comparing the AUCs. Correlation of the new marker and the existing marker, conditional on the outcome, is represented by ρ.

We repeated our analyses varying the prevalence (0.2 and 0.05) and sample size (n=100). Our results were essentially unaffected. Lowering the sample size or prevalence reduced power for all analyses, but the Wald and likelihood ratio tests always had far superior power to the AUC test.

## Summary

We have shown that a test of a new marker in a prediction regression model and a test of prediction accuracy constructed from a comparison of ROC AUCs are designed to test the same conceptual null hypothesis, the hypothesis that the new marker possesses no incremental predictive information. The use of two tests side by side to address the same hypothesis is not logical, and if we require both tests to be significant such a strategy necessarily has the effect of decreasing the power for detecting an improvement in predictive accuracy. We have also shown that testing ROC areas generated from nested models is an approach with serious validity problems.

What is the correct approach? In our opinion, investigators should select a single test for assessing incremental predictive accuracy. The choice of test depends on the data context, but tests of the incremental predictive information in the new marker in the context of the regression model, such as the likelihood ratio test and the Wald test, have well understood and valid statistical properties under the defined sampling properties of the model. Likewise, the area test of Delong et al. [8] has been shown to have valid statistical properties in the context for which it was developed, that is, the comparison of two dependent diagnostic markers[17]. But ROC analysis was not developed for the purpose of testing new predictors in nested models. ROC analysis was created as a technique to appropriately calibrate comparisons of the accuracy of two or more ordinal diagnostic procedures [18]. In this setting, where the empirical classification points for each of the tests are not under the control of the analyst, ROC methods are needed to extrapolate the results of each of the diagnostic (or predictive) tests to permit a calibrated comparison. The use of ROC AUCs is a natural outgrowth of this work, in that the AUC is summary measure that is inherently calibrated for comparison purposes [19]. Subsequent work generalized these methods to incorporate comparisons of continuous (rather than ordinal) tests, such as two distinct laboratory tests[20].

However, we have shown that ROC methods are not appropriate for testing the incremental value of new predictors in the presence of established predictors (i.e. in a "nested" setting) where the patient-specific predictors from the model are used as the data for the ROC analysis. There are several possible reasons why the area test is so inferior in this context. Our intuition is that a principal reason is that the use of patient-specific predictors from the estimated model as data ensures that the estimated ROC curve is biased upwards [21]. This is a well-known phenomenon that had led to the wide-spread recognition that predictive models need to be validated in independent test sets, or minimally by using cross-validation techniques. Since the bias is strongly correlated in the two nested models the validity of the test is further compromised.

Naturally, novel ROC methods might be developed that overcome the problems associated with the De Long et al. test in this context. Such novel methods could be formally evaluated in future research. Nonetheless, the validity or otherwise of these methods would not affect our main conclusion that using both an ROC test and a test of a new marker in a predictive model is double-testing the same conceptual null hypothesis and that, accordingly, investigators should select a single test for assessing incremental predictive accuracy.

Several previous authors have pointed out that AUC comparisons are underpowered. Pencina, for example, states that "very large 'independent' associations of the new marker with the outcome are required to result in a meaningfully larger AUC"[22]. Indeed, this observation was the motivation for the novel 'net reclassification index' method. Similarly, in a comprehensive review, Cook argued that the AUC is "insensitive" and "can erroneously eliminate important clinical risk predictors"[23]. These considerations appear to be based largely on practical experience and case studies. Previous authors made no formal investigations of the statistical properties of area tests nor documented clearly that comparison of ROC areas for nested models is highly conservative. We believe that the huge power and size deficiencies of the AUC test in this context, demonstrated in our simulations, are not widely known to methodologists.

The ROC curve, a descriptive tool, adds insight in that it allows one to identify and compare the sensitivities of the predictive models at chosen specificities, and vice versa, and for gauging the magnitude of any apparent increase in accuracy. ROC curves are useful for characterizing the predictive accuracy of competing predictive models, even nested models, as numerous commentators have advocated. But its role in this setting is descriptive, and it is imperative that cross-validated data or independent data from external data sets be used for this purpose. Elsewhere it has been argued that ROC curves are inadequate for assessing prediction models, including the question of whether new markers are informative [6,24]. Simple, decision analytic approaches have also been advocated [25-27]. Such positions are not inconsistent with our advice here. We suggest a staged approach, with analysis of the value of a new predictor first involving assessment of independent statistical significance of the predictor within a predictive model, then increment in AUC and, finally, impact on decision making[6].

In summary, we advise against the use of double-testing new predictors for predictive accuracy in the presence of existing predictors. This strategy involves essentially testing the same conceptual null hypothesis twice, disrupting the validity of the reported significance levels. Further, the use of tests based on ROC curves generated from the estimated patient-specific risk predictors without cross-validation has been shown to be technically flawed. However, ROC curves are useful as tools for describing the rate of false positive and false negative errors.

## Abbreviations

ROC: Receiver operating characteristic curve; AUC: Area under the curve

## Competing interests

The authors declare that they have no competing interests.

## Authors' contributions

The study was conceived and written by AJV and CBB. AMC conducted the simulation studies. All authors read and approved the final manuscript.

## Appendix

### Simulation approach

#### Independent Predictors

Simulations were conducted to test the power of the likelihood ratio test, the Wald test and a comparison of areas-under-the-curves. In this first set of simulations, *X *and X* are independent normal random variables. Among subjects who experience the outcome (y=1), *X *has a normal distribution with mean μ and variance 1, and X* has mean μ* and variance 1. Among non-responders, x and x* are both drawn independently from a standard N(0,1) distribution. We first determine π, the overall probability of response (which was set at 0.5 in all our simulations), and n, the total sample size (which was set at 500).

##### Data Generation

For each of n patients generate data as follows:-

1. Generate y: Sample from U(0,1), set y=1 if U(0,1)<π and set y=0 otherwise.

2. If y=1, sample X from N(μ,1) and X* from N(μ*, 1).

3. If y=0, sample X from N(0,1) and X* from N(0, 1).

##### Data Analysis

4. Analyze the restricted model using the logistic regression logit(y) = *β*_*0 *_*+ β*_*1*_*X*

5. Use the estimates of β_0 _and β_1 _from the model to calculate $z={\overset{\wedge }{\beta }}_{0}+{\overset{\wedge }{\beta }}_{1}X$. Note that the true value of *β*_*1 *_= μ and *β*_*0 *_*= *0.5 μ^2 ^+log(π ÷ (1-π)).

6. Calculate the area under the ROC curve linking y and z

7. Analyze the expanded model using the logistic regression logit(y) = β_0 _+ *β*_*1*_*X *+ *β*_*2*_X*

8. Calculate a p-value for H: β_2 _= 0 using the likelihood ratio test and the Wald test.

9. Use the estimates of β_0_, β_1 _and β_2 _from the model to calculate $z={\overset{\wedge }{\beta }}_{0}+{\overset{\wedge }{\beta }}_{1}X+{\overset{\wedge }{\beta }}_{2}X*$

10. Calculate the area under the ROC curve linking y and z

11. Calculate the DeLong et al. p-value comparing the area estimates from 6 and 10 above.

#### Simulation

12. Repeat the entire process from 1-11 above 2000 times and compute the relative frequency of significant (P < 0.05) p-values.

13. Do this for μ*=0 to estimate the test size, and for larger values of μ* to assess a range of powers.

14. Repeat the process for a range of values of μ.

#### Dependent Predictors

In this dependent setting, among subjects who experience the outcome (y=1), x and x* are both normally distributed with unconditional variances equal to 1, means μ and μ* respectively, and with correlation ρ. Among non-responders, the means of both *X *and X* are zero and the correlation is ρ.

##### Data Generation

For each of n patients generate data as follows:-

1. Generate y: Sample from U(0,1), set y=1 if U(0,1)<π and set y=0 otherwise.

2. If y=1, sample *X *from N(μ,1) and X* from N(μ*+*ρX*,1-p^2^).

3. If y=0, sample *X *from N(0,1) and X* from N(*ρX*, 1-p^2^).

##### Data Analysis

4. Analyze the restricted model using the logistic regression logit(y) = β_0 _+ *β*_*1*_*X*

5. Use the estimates of β_0 _and β_1 _from the model to calculate $z={\overset{\wedge }{\beta }}_{0}+{\overset{\wedge }{\beta }}_{1}X$

6. Calculate the area under the ROC curve linking y and z

7. Analyze the expanded model using the logistic regression logit(y) = β_0 _+ *β*_*1*_*X *+ *β*_*2*_X*

8. Calculate a p-value for H: β_2 _= 0 using the likelihood ratio test and the Wald test.

9. Use the estimates of β_0_, β_1 _and β_2 _from the model to calculate $z={\overset{\wedge }{\beta }}_{0}+{\overset{\wedge }{\beta }}_{1}X+{\overset{\wedge }{\beta }}_{2}X*$

10. Calculate the area under the ROC curve linking y and z

11. Calculate the DeLong et al. p-value comparing the area estimates from 6 and 10 above.

### Simulation

12. Repeat the entire process from 1-11 above and compute the relative frequency of significant (P < 0.05) p-values.

13. Do this for μ*=0 to estimate the test size, and for larger values of μ* to assess a range of powers.

14. Repeat the process for a range of values of μ.

## Pre-publication history

The pre-publication history for this paper can be accessed here:

http://www.biomedcentral.com/1471-2288/11/13/prepub
